# Client satisfaction in a faith-based health network: findings from a survey in Uganda

**DOI:** 10.4314/ahs.v17i3.38

**Published:** 2017-09

**Authors:** Constance Sibongile Shumba, Kenneth Kabali, Jonathan Miyonga, Jairus Mugadu, Luke Lakidi, Patrick Kerchan, Tonny Tumwesigye

**Affiliations:** Uganda Protestant Medical Bureau, 877 Balintuma Road, Mengo, Kampala, Uganda

**Keywords:** Client satisfaction, faith-based health facilities, health services, quality improvement, Uganda

## Abstract

**Background:**

Client satisfaction surveys are important in evaluating quality of the healthcare processes and contribute to health service improvements by assisting health program managers to develop appropriate strategies. The goal of this study was to assess clients' level of satisfaction with services provided by private-not-for-profit member health facilities affiliated to Uganda Protestant Medical Bureau.

**Methods:**

This was a cross-sectional descriptive study using an interviewer-administered questionnaire conducted in 254/278 (91%) of UPMB member health facilities between 27th April and 14th July 2014 among 927 clients. The tool measured ten dimensions of the care-seeking experience namely; health facility access; waiting time; health providers; support staff; rights; payments; facilities and environment; consent; confidentiality; and the overall care seeking experience. Logistic regression was utilised for multivariate analysis.

**Results:**

Overall client satisfaction was found to be high within the UPMB network (84.2%). Most of the client satisfaction dimensions were rated above 70% except payments and rights. There was evidence of association with marital status; single/never married were 3.05 times more likely to be dissatisfied compared to widowed. Clients attending HCIII were less likely to be dissatisfied compared to those attending HCII (OR=0.51, 95% CI: 0.25–1.05). Post-secondary education (OR=1.79; 95% CI 1.01–3.17), being formally employed (OR=2.78, 95% CI: 0.91–8.48) or unemployed (OR=3.34, 95% CI: 1.00–11.17), attendance at a hospital (OR=2.15, 95% CI: 1.36– 3.41) were also associated with high dissatisfaction levels with payments.

**Conclusion:**

This study found a high level of satisfaction with services in the UPMB network but recorded low client satisfaction with the dimensions of rights and payments. Health workers should take time to explain rights and entitlement as well as charges levied to clients.

## Introduction

Client satisfaction is a vital part of the healthcare process and signifies the relationship between clients' expectations and the actual service experience^1^,^2^. When the experience closely matches the expectation, then satisfaction is likely to be high and this will have a positive impact on the health seeking behavior, adherence to treatment recommendations and other health advice.^1^–^4^ In low-income countries where consumer protection groups are few or in some cases non-existent, clients may not verbally express their dissatisfaction to healthcare providers and resort to traditional or other alternative forms of care^1^. Client satisfaction surveys are therefore important for evaluating quality of care^5^. Client satisfaction surveys present an opportunity for clients to express their perception on health service provision and assist providers and policy makers in identifying gaps and strategies in health service delivery^3^,^6^. The scaling-up of various interventions in the healthcare setting including HIV care and treatment in many low-income settings such as Uganda has given rise to several quality concerns^6^.

Client satisfaction rates have been found to be between 40% and 74.6% in other studies conducted in Uganda. There is a paucity of studies on client satisfaction in both the private and public health sector in Uganda^5^,^7^–^11^. Some of these few studies were limited in that they measured satisfaction for particular health services or programs^9^–^11^. There are no surveys recorded within the Uganda Protestant Medical Bureau (UPMB), a faith-based network of 278 health facilities.

The goal of this study was to assess clients' level of satisfaction with services provided by Private-Not-For-Profit (PNFP) member health facilities under Uganda Protestant Medical Bureau (UPMB). The study was conducted due to a knowledge gap on client satisfaction with healthcare services within the UPMB network where such an undertaking had not been done before. This was particularly important in view of the growing recognition of the role of quality improvement and concerns about patient safety in healthcare. The Uganda Health Sector Strategic and Investment Plan (2011–14) had a specific interest in exploring and deepening understanding of client and organizational characteristics linked to client satisfaction^12^. The findings are useful in designing and implementing service improvement plans in the network health facilities.

## Methodology

### Study setting

UPMB was founded in 1957 as a charitable, faith-based non-governmental, national umbrella organization for the disbursement of grants in aid to Protestant mission hospitals and to serve as a liaison between Government of Uganda, donors and member health facilities. In 2014, UPMB was supporting the activities of 278 health units affiliated to the Church of Uganda, Adventist and Pentecostal churches of Uganda. The 278 units comprise 18 hospitals with 10 Health Training Institutions, 6 Health Centre IVs, 254 Health Centre IIIs and IIs which is the lowest level of a physical health facility that treats minor illnesses. The health facilities form about 35% of the private-not-for-profit health facilities across Uganda. Approximately 80% of the health facilities are located in rural, poor and post-conflict communities representing an important social asset for the communities, and have grown out of initiatives of congregations to address identified need.

### Study design

This was a cross-sectional descriptive quantitative study using an interviewer administered questionnaire conducted in 254/278 (91%) of UPMB member health facilities from April to July 2014. The client satisfaction tool was designed based on a literature review of existing tools, and was piloted in five central region health facilities in Kampala and Wakiso districts. The tool measured ten dimensions of the care-seeking experience namely; Ease of accessing the health facility; waiting time; health providers; support staff; rights; payments; facilities and environment; consent; confidentiality; and the overall care seeking experience.

### Sample size and sampling method

In order to assess clients' satisfaction with the services provided by UPMB's member health facilities, interviews were conducted with a total of 927 clients who sought health services at UPMB network health facilities. The sampling unit was Hospitals, HCIVs, HCIIs and hciis in the network. The out-patient and in-patients of health facilities visited on the day of the survey constituted the sampling frame. The researchers took a 30% representative sample for all out-patient department (OPD) client contacts seen in a day in the network to generate the desired power for the study. Based on the 2012–2013 UPMB network OPD contacts, that is, 1,440,741 the daily contact was 3,947 clients per day (1,440,741 annual clients/365 days a year) in the network bringing the 30% representative sample to 1,184 clients. Probability-proportional-to-size sampling based on daily patient load and facility level was used and the sample was spread by level of health facility as shown in the table below to give the number that was interviewed at every facility. Different hospital departments were purposively sampled and clients randomly selected from these departments. Table 1 shows the sampling criteria for the client satisfaction survey.

**Table 1:** Sampling Criteria for Client Satisfaction Survey

Level of care	2012–13 Report rate	OPD contacts	With 100% Reporting (n)	(n/365)	X= 30% of (n/365)	No of facilities Y	No. Clients to interview per facility X/Y
**Hospital**	100%	452,045	452,045	1,238	372	18	21
**HC IV**	100%	63,121	63,121	173	52	7	7
**HC III**	90.6%	775,632	925,575	2,536	761	252	3
**HC II**	82%						
**Total**			**1,440,741**	**3,947**	1,184	277	

### Data collection procedures

Data collection tools were designed and reviewed by UPMB staff who also conducted the field data collection. A short interviewer-administered questionnaire was used to conduct these interviews. The tool was designed using a Likert scale (5-excellent; 4- good; 3- fair; 2- poor; 1- very poor).

### Data management and analysis

Data was entered using Epidata software and analyzed using STATA version 12 and SAS. Clients who rated the services as excellent or good were classified as satisfied while those who rated very poor, poor, fair were classified as dissatisfied. Chi-square test was used to find the relationship between categorical variables. The distribution of participants' characteristics and satisfaction levels were analyzed at univariate analysis. To establish the relationship between clients' satisfaction and other independent variables (demographics, health facility level, diocese among others), logistic regression was performed giving results as odds ratios. The level of significance was set at 0.05 with 95% confidence interval.

### Ethical considerations

This study was done as part of an operational research activity for the UPMB strategic plan 2014–18 baseline assessment with ethical approval from the Uganda National Council for Science and Technology (SS3823). Participants were informed that their participation was voluntary and they had a right to withdraw from the interview at any time. No names were recorded during the interviews and only codes were used to identify participants.

## Results

### Socio-demographic characteristics of clients

In total, 927 clients were interviewed with a response rate of 78.3%. More than one third of those interviewed were aged 19–30 (35.8%), while those aged 31–40 made up 14.7% and 9.5% were 51 years or older. Majority (63.5%) were married or cohabiting, 39.0% of the clients had primary education as the highest level of educational attainment and 19.6% had no formal education at all. Over half (50.4%) of the respondents were peasant farmers. Most (65.0%) of the respondents were Protestants (Table 2).

**Table 2:** Socio-Demographic Characteristics of respondents

Variable	Percentage (%)	Number (N=927)
Age of clients		
0–18	11.6%	108
19–30	35.8%	332
31–40	14.7%	136
41–50	8.3%	77
51+	9.5%	88
Marital status		
Single/never married	26.8%	248
Married/cohabiting	63.5%	589
Divorced/separated	4.4%	41
Widowed	5.3%	49
Highest level of education attained		
No education	19.6%	182
Primary	39.2%	363
Secondary	27.9%	259
Post-secondary	13.3%	123
Occupation		
Not employed	6.8%	63
Peasant farmer	50.4%	467
Trader/business	13.3%	123
Occupation		
Not employed	6.8%	63
Peasant farmer	50.4%	467
Trader/business	13.3%	123
Formal employment	9.0%	83
Technical/craft	2.6%	24
Other	18.0%	167
Religious affiliation		
Protestant/SDA/Anglican/born again	65.0%	603
Catholic	24.7%	229
Muslim	7.6%	70
Other	2.7%	25

^1^Lowest overall satisfaction rate

^2^Lowest rating on payment dimension

^3^Lowest rating on rights dimension

** Factors shown in univariate model had LRT p-value < 0.10

* Wide confidence interval due to small sample size

### Type of services sought by clients

**Figure 1:**
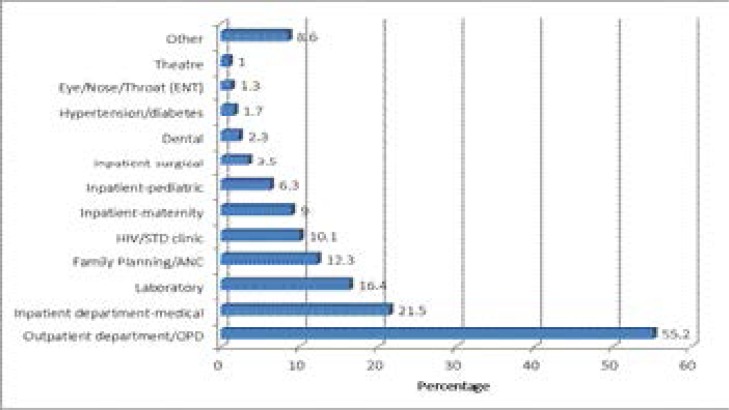
Type of services sought by clients on interview day.

Most (55.2%) of the clients interviewed sought services from the outpatients department on the interview days. In-patients were also interviewed; 21.5% in medical, 9% in maternity, 6.3% in pediatric, and 3.5% in surgical department. There were also a substantial number of clients that had sought laboratory services (16.4%), family planning and ANC services (12.3%), and HIV/STI services (10.1%) as shown in Figure 1.

### Distance between Client's home and the Health Facility visited

The majority of clients' (64.0%), lived between 0–4 kms from their homes to the facility they visited on the day of interview, 15.7% between 5–9kms, 5.2% between 10–14 kms, 2.5% had travelled between 15–19 kms to reach the health facility. Notably, 12.6% had travelled 20 kms or more to reach the health facilities they visited. For 46% of clients (N=426), there was a nearer health facility to their home than the one they visited at the day they were interviewed. Reasons given for not visiting the nearest facility were; overall poor quality of services, (18.7%), unavailability of drugs (18.4%), unavailability of staff (10.2%), cost (6.7%).

Satisfaction with the overall level care seeking experiences was 84.2% (Table 3). For most of the client satisfaction dimensions, there was a satisfaction rate of over 70.0%. This pattern was consistent for almost all dimensions. However, confidentiality was rated highest at 90.8%. The lowest client satisfaction ratings were for the dimensions relating to rights and payments. The amount charged and the explanation of charges provided were particularly identified as issues of dissatisfaction. In addition, clients felt that they were not fully made aware of their rights and entitlements and were not satisfied that they were facilitated with information to make choices and decisions that suited them.

**Table 3:** Client satisfaction with the different care dimensions assessed.

Client satisfaction dimension	Satisfied
**Ease of accessing the health facility**	84.8%
**Waiting time**	*78.5%*
**Health providers**	*77.8%*
**Support staff**	*73.9%*
**Rights**	*63.0%*
**Payments**	*64.7%*
**Facility**	*72.4%*
**Consent**	*77.2%*
**Confidentiality**	*90.8%*
**Overall care seeking experience**	*84.2%*

Overall satisfaction increased with age up to the age 40 and reduced beyond this age. The single/never married had lower satisfaction levels compared to the married, widowed and divorced. Satisfaction levels were generally lower in Health Centre IV and hospitals compared to the lower level centres. In regard to satisfaction of payment for services, females were more satisfied than males, satisfaction levels increased with education but dropped after secondary education to a level below those with no education. Those in formal employment and the unemployed had the lowest levels of satisfaction for payment (<50%) while satisfaction levels were fairly the same across all other occupational groups. Protestants and Catholics had lower satisfaction with payments compared to the Muslims and other religion. In terms of rights, the divorced had lower levels of satisfaction compared to the widowed, married and never married/single. Muslims had a lower rate of satisfaction with the rights dimension compared to Protestants, Catholics and other religions (Table 4).

**Table 4:** Association between client satisfaction and demographic characteristics

Satisfaction dimension	Factors	Satisfaction N (%)	p-value of Chi-square
1. Overall satisfaction	**Age**		
	0–18	79 (73.2%)	0.078
	19–30	332 (84.5%)	
	31–40	136 (80.0%)	
	41–50	77 (81.9%)	
	51+	88 (86.3%)	
	**Marital status**		
	Single/Never married	188 (78.7%)	0.033
	Married	484 (84.3%)	
	Widowed	43 (91.5%)	
	Divorced	36 (92.3%)	
	**Level of care**		
	Health Centre II	307 (85.8%)	0.0094
	Health Centre III	121 (88.3%)	
	Health Centre IV	56 (72.7%)	
	Hospital	183 (80.6%)	
2. Satisfaction with Payments	**Sex**		
	Male	147 (55.3%)	0.055
	Female	348 (62.3%)	
	**Education**		
	No education	82 (52.9%)	0.001
	Primary education	218 (66.9%)	
	Secondary	142 (60.9%)	
	Post-secondary	53 (47.8%)	
	**Occupation**		
	Not employed	18 (36.0%)	0.001
	Peasant farmer	265 (64.3%)	
	Trader/business	66 (60.0%)	
	Formally employed	38 (48.7%)	
	Technician/craft	15 (68.2%)	
	Other	93 (60.8%)	
	**Religion**		0.087
	Protestant	317 (58.5%)	
	Catholic	122 (58.9%)	
	Muslim	44 (73.3%)	
	Other	12 (75.0%)	
3. Satisfaction with rights	**Marital status**		
	Single/Never married	141 (64.7%)	0.066
	Married	311 (61.3%)	
	Widowed	31 (75.6%)	
	Divorced	16 (47.1%)	
	**Religion**		
	Protestant	319 (60.3%)	0.079
	Catholic	137 (68.8%)	
	Muslim	31 (55.5%)	
	Other	12 (75.0%)	

Across all the three dimensions, diocese was associated with satisfaction although this varied (Table 5).

**Table 5:** Overall client satisfaction, satisfaction with payments and rights by Diocese

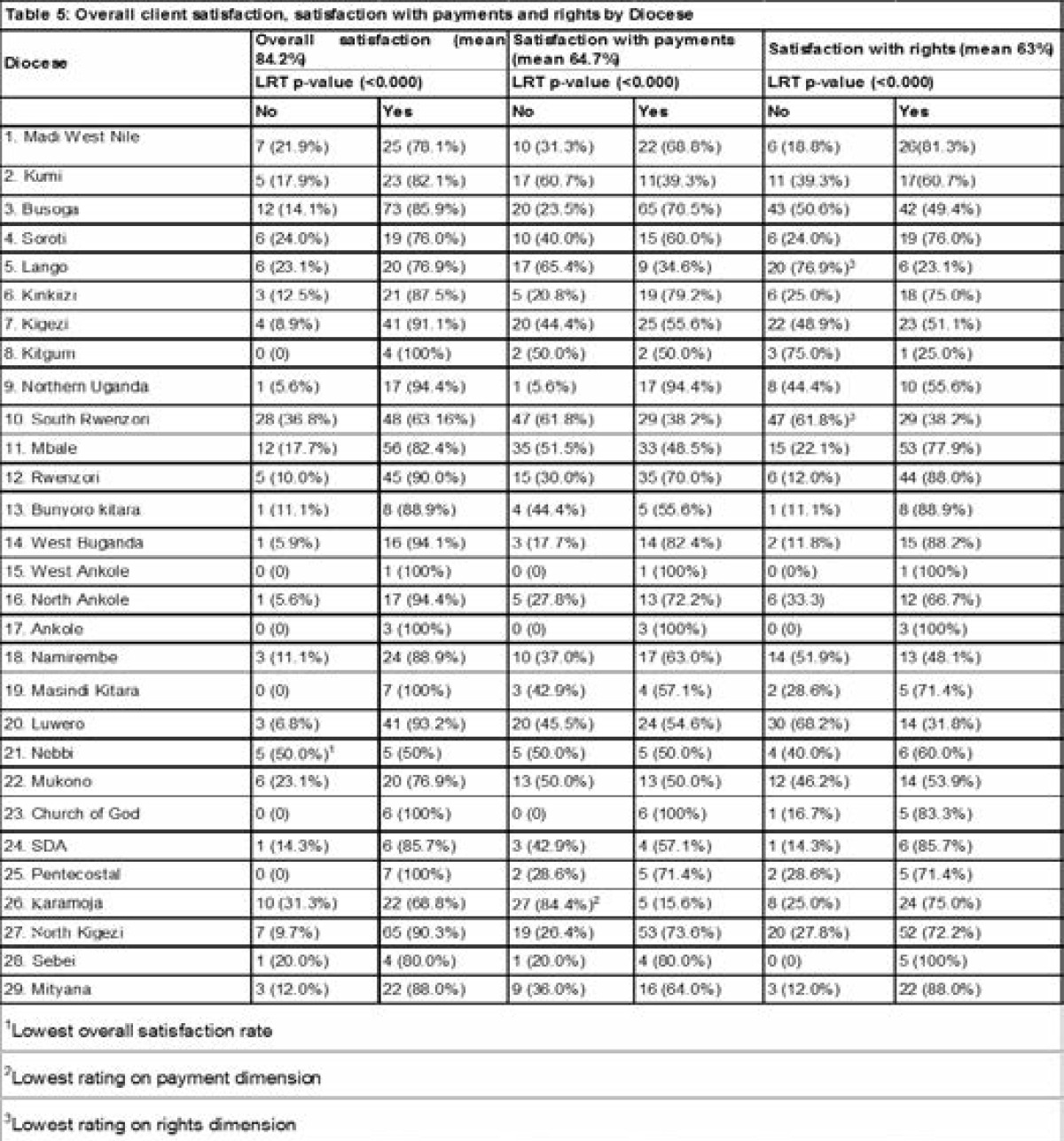

A diocese is an administrative unit under the stewardship of a bishop within the church structure and consists of parishes. The units relating to the diocese do not necessarily correspond to the government district administrative structures and in Uganda, most of the dioceses combine two or more of the government administrative districts. The Church of Uganda has 35 dioceses 13 and as the technical health arm of the Church of Uganda, SDA and other Protestant churches, UPMB works within the diocese structures in terms of defining stewardship of the member health facilities majority of whom are founded by the Church of Uganda. Therefore in this study, the dioceses' correspond to a group of protestant church-founded health facilities within the administrative units and are members of UPMB. For the other protestant churches that do not have dioceses, the study grouped these by the church affiliation.

The following dioceses had low overall satisfaction rates; South Rwenzori, Nebbi, Karamoja, Mukono and Lango. In regard to satisfaction with the payments dimension; Kumi, Lango, South Rwenzori, Mbale, Mukono, Kigezi, Karamoja and Luwero diocese had lower satisfaction rates. Concerning the rights dimension; Busoga, lango, Kigezi, kitgum, South Rwenzori, Namirembe, Luwero and Mukono dioceses had lower satisfaction. In general, the following dioceses had high dissatisfaction across all the satisfaction dimensions (overall satisfaction, payment, rights); South Rwenzori, Mukono and Lango. Table 6 shows the factors associated with client dissatisfaction with health services in UPMB facilities.

**Table:** 

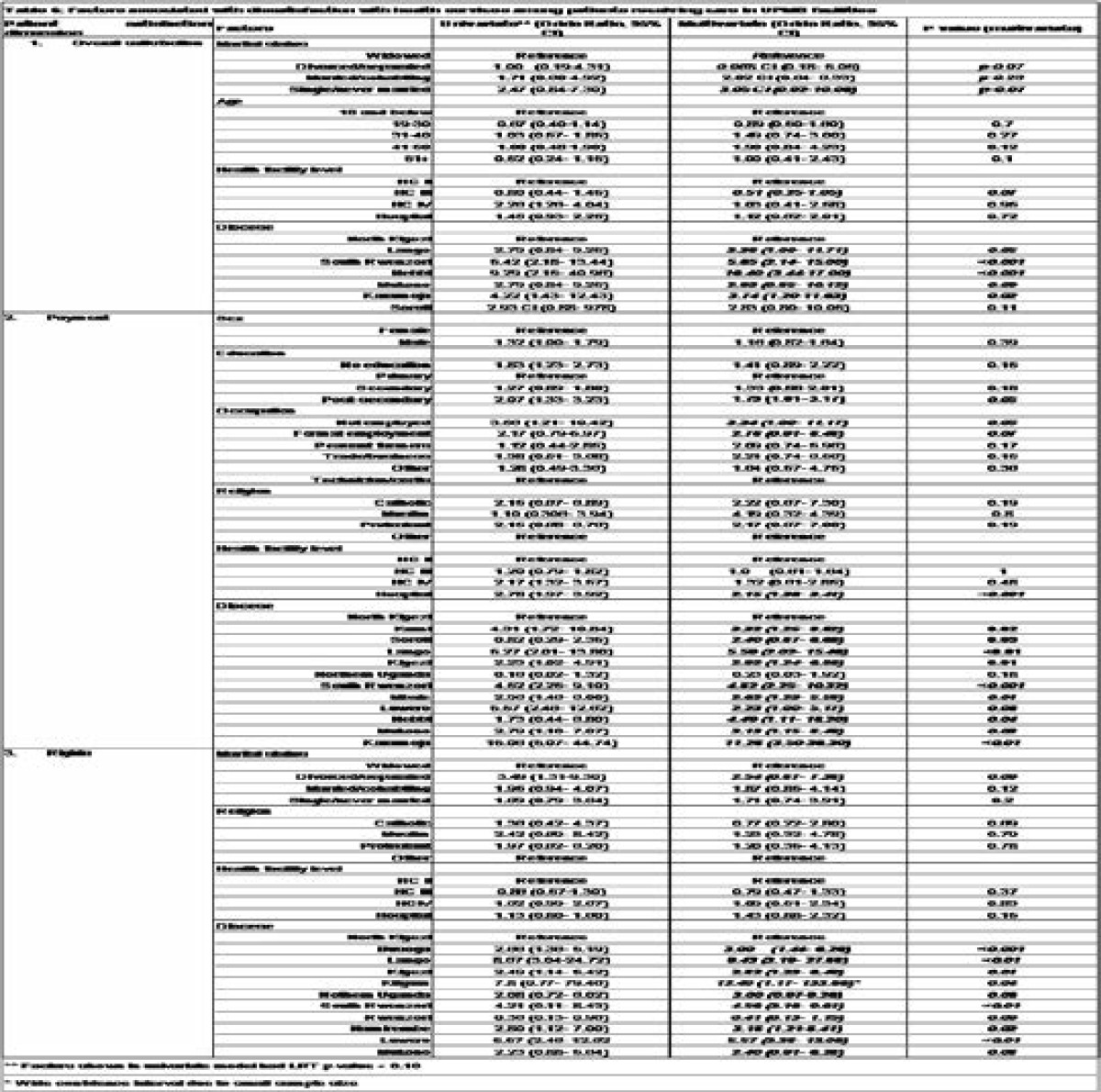

### Overall satisfaction

In the multivariate analysis the patients' dioceses were significantly associated with overall dissatisfaction. The dioceses of Nebbi (OR=16.4), South Rwenzori (OR=5.65), Karamoja (OR=3.74), Lango (OR=3.38), Mukono (OR=2.93) had higher levels of dissatisfaction compared to North Kigezi. There was some evidence of association with marital status, single/never married were 3.05 times more likely to be dissatisfied compared to widowed. Clients attending HCIII were less likely to be dissatisfied compared to HCII (OR=0.51). The association with age observed in the univariate model disappeared in the final model.

### Dissatisfaction with payments dimension

The dioceses association with dissatisfaction was also observed in payments but with a different set of dioceses added to Nebbi (OR=4.49), South Rwenzori (OR=4.82), Karamoja (OR=11.28), Lango (OR=5.59) and Mukono (OR=3.13); Kumi (OR=3.32), Soroti (OR=2.40), Kigezi (OR=2.92), South Rwenzori (OR=4.48), Mbale (OR=2.62), Luwero (OR=2.23) were the additional dioceses where clients showed higher odds of dissatisfaction compared to those in North Kigezi. Post-secondary education (OR=1.79), being formally employed (OR=2.78) or unemployed (OR=3.34), attendance at a hospital (OR=2.15) were also associated with high dissatisfaction levels with payments. Religion and gender were only associated with dissatisfaction at the univariate analysis.

### Dissatisfaction with the rights dimension

In addition to South Rwenzori (OR=4.56), Mukono (OR=2.40) and Lango (OR=9.43), the patients in the following dioceses also showed higher odds of dissatisfaction with the rights dimension; Busoga (OR=3.00), Kigezi (OR=2.82), Kitgum (OR=12.40), Northern Uganda (OR= 3.00), Namirembe (OR=3.18), and Luwero (OR=5.57) compared to North Kigezi. Clients from Rwenzori were less likely to be dissatisfied compared to North Kigezi. There was some evidence of association with marital status, as the divorced/separated had higher odds of dissatisfaction (OR=2.54) in respect to rights compared to the widowed.

## Discussion

The study found an overall client satisfaction level of 84.2%. While few studies have been conducted to assess satisfaction levels in Uganda, a study to assess the quality of antenatal care services in Eastern Uganda, reported overall satisfaction rate of 74.6%^11^. This was attributed to clients having sought care in public facilities with high patient volumes and frequent stock outs of medicines in contrast to private-not-for-profit facilities as in our study. Other studies in Uganda reported much lower satisfaction levels. For example, a study conducted in 10 districts in 2013 found a client satisfaction rate of 47%^8^. Another study conducted in two facilities providing ART services, a public health facility and a private health facility in Kabale, found that satisfaction was 58% and 64% respectively^10^. A study on satisfaction among clients attending eye clinics in Masaka, Uganda found a low (40%) satisfaction rate^9^. Our findings are only comparable to findings in Nigeria where overall satisfaction was found to be 83%^1^. A much higher rate of overall satisfaction of 94.7% was found in Nigeria^2^. However, these studies were conducted in a different context. A review on faith-inspired services in African countries including Uganda found that they had significantly higher satisfaction than public services^14^.

This study also found that marital status, health facility level and dioceses were associated with overall satisfaction. The single/never married were more likely to be dissatisfied with the overall service experience while dissatisfaction among clients at Health centre III was lower showing that the clients interviewed were generally satisfied with the overall service experience at that level. The clients' dissatisfaction experienced at Hospitals and Health centre IVs could be related to a high patient volume in comparison to lower level centres. High patient volumes often lead to long waiting times and a short clinician-patient interaction time. The dioceses of Nebbi, South Rwenzori, Karamoja, Lango and Mukono had higher client dissatisfaction rates suggesting the need to improve on the different client satisfaction dimensions.

The mean satisfaction rating in our study for payments was considerably low at 64.7% as clients complained that payments were not clear. This is similar to the study in Mulago Hospital in Uganda and one in Masaka where cost of health services was associated with low satisfaction^5^,^9^. This could be due to the fact that most clients may not be aware of the out-of-pocket payments required for different services and explaining this before hand in informational sessions may improve clarity^5^. The level of out-of-pocket expenditure in Uganda stood at 33% in 2014 and for health facilities in the UPMB network, user fees contributed to 41% of the income for recurrent operations^15^,^16^. Therefore, it is not surprising that there was low satisfaction with the payments and while the goal is to keep charges as low as possible, the decrease in government contribution to the PNFP sector means that there is a growing dependence on user fees for member health facilities. In contrast the study in a teaching hospital in Northern Nigeria found a higher (73%) rate of satisfaction with the payment for services provided among the respondents^1^.

There was a higher dissatisfaction with payments in hospitals compared with Health Centre IIs and this could indicate higher user fees at hospital level due to more complicated illnesses that require more procedures, high opportunity costs such as transport in order to access health care in hospitals or high costs for procedures due to the need to meet the overall operating costs such as remunerating specialists. The unemployed were significantly dissatisfied with payments and this is not surprising given the lack of income. Interestingly though, the formally employed were also dissatisfied with the payments for services provided indicating that they may perceive that there is low value for money for services rendered^5^. Additionally, the formally employed might be experiencing a huge burden of paying for the costs of health care for themselves and their dependents thus taking a large share of their household incomes. The study also found that those with post-secondary education were also dissatisfied with payments. Those with post-secondary education are likely to be also formally employed and the same reasons for this might be applicable. Similarly, education level, and estimated expenditure were associated with the mean general satisfaction scores in outpatient clinics in Mulago Hospital in Uganda^5^. The findings on payment dissatisfaction should inform exemption policies since the unemployed are not satisfied with charges and this could be due to their lack of income.

The dioceses were also associated with high payment dissatisfaction in our study suggesting that there may be different user fee structures across dioceses. Alternatively, the dissatisfaction in the diocese may reflect the variations in incomes across the dioceses and the lack of sensitivity of user fees to these. This may have a negative impact on equity and access to health care. It is important for the different dioceses to consider the socio-economic standing of clients when setting user fees. In addition it may be important for the network to advocate for more budgetary support for facilities in dioceses where clients are unable to afford access to health care.

The overall satisfaction rate with the dimension of rights in our study was 63% and this is similar to a study conducted in Portugal which also found a 63% rate of satisfaction with rights awareness^17^. This signaled dissatisfaction of clients in the way health providers engaged with them in informing them of their entitlements and in the decision-making process. In this study, marital status and dioceses were significantly associated with higher dissatisfaction and those who were divorced or separated where more likely to be dissatisfied with respect for their rights as patients. Similarly, marital status has been linked to consciousness of client rights^18^. Client rights are aimed at protecting their autonomy and while health providers may have an understanding of this, client rights are not always upheld. Information on rights and entitlements is not always fully given leading to dissatisfaction^17^. This is so in spite of the need to give personalized information that allows patients to make informed choices that suit them in their circumstances and improve their quality of life. Health providers need to recognize that the client-provider relationship has evolved over the years from one of paternalism where the provider knows it all to one where there is a strong focus on human rights and individual autonomy and curiosity is encouraged enabling effective participation in decision-making^19^.

Client satisfaction improves a health facility's image and can result in high service uptake^18^. Furthermore, when patients are kept aware of their care using simple language that is easy to understand, this alleviates anxiety and increases satisfaction. Client rights refers to the operationalization of human rights in health care enabling clients to get appropriate and respectable care according to need based on the premise of preserving human dignity^20^. In Uganda, there is a patients' charter which spells the client rights and responsibilities as well as the responsibilities of the health workers and is aimed at increasing clients' awareness of their rights and encourage them to demand good quality health services^21^. This charter was introduced against the backdrop of limited capacity to demand health rights by patients in the country.

In this study, 46% of clients had a health facility that was closer to their home than the one they visited on the day they were interviewed. While clients' physical proximity to their preferred health facility could indicate the level of access to health care, this study revealed that for close to half of the clients, the nearest health facility was not necessarily the one they used for different reasons. The quality of services, availability of drugs and availability of staff were found to be key determinants of clients' choice of health care sources. This conforms to other findings where the same factors were also found to influence access to health care^14^,^22^. It is therefore not sufficient to guarantee physical proximity only, as clients will leave nearer facilities to look for those with better care, even if far away. All efforts should therefore be made to improve the quality of care available at all health facilities particularly those in dioceses with greater dissatisfaction rates. Most importantly, the dioceses' that had high dissatisfaction across all the satisfaction dimensions (overall satisfaction, payment, rights) namely South Rwenzori, Mukono and Lango need urgent attention to be able to improve the service experiences of clients.

Our study was conducted in a heterogeneous population spread geographically across all the different regions of the country and at different health facility levels. The study was conducted only in the private-not-for-profit protestant faith-based network therefore may not be generalizable to the public sector health facilities. Due to the cross-sectional nature of this study, it is important to interpret the findings with caution as satisfaction may vary with different service encounters and is subject to extrinsic and peculiar factors unrelated to the service experience^23^. In addition clients' satisfaction assessments may be subjective.

This study indicates a high level of satisfaction with services in the UPMB faith-based network in Uganda. The study has also highlighted significant dissatisfaction with the payment and rights dimensions. This has some implications for service delivery within the network. The findings suggest the need to develop service improvement plans to address concerns around overall client satisfaction, payments and rights. There is need to standardize and explain the different charges levied on clients. To reduce out-of-pocket expenditure, there is need to implement community health insurance schemes. Health facility managers should ensure that the number of health workers match patient volumes especially at higher level centres. Health workers should take more time to explain rights and entitlement to clients as part of health education talks. All member health facilities should be encouraged to conduct routine client satisfaction surveys and implement continuous quality improvement strategies. Training of health workers on the patient charter and provision of job aids is necessary to promote patient-centered care in UPMB facilities.

## References

[1] Iliyasu Z, Abubaker IS, Abubaker S, Lawan UM, Gajida AU (2010). Patients' satisfaction with services obtained from Aminu Kano Teaching Hospital, Kano, Northern Nigeria. Nigerian Journal of Clinical Practice.

[2] Ogunfowokan O, Mora M (2012). Time, expectation and satisfaction: Patients' experience at National Hospital Abuja, Nigeria. Afr J Prm Health Care Fam Med.

[3] Babikako HM, Neuhauser D, Katamba A, Mupe E (2011). Patient satisfaction, feasibility and reliability of satisfaction questionnaire among patients with pulmonary tuberculosis in urban Uganda: a cross-sectional study. Health Research Policy and Systems.

[4] Rajbanshi L, Dungana GP, Gurung YK, Koirala D (2014). Satisfaction with health care services of outpatient department at Chitwan Medical College Teaching Hospital, Nepal. Journal of Chitwan Medical College.

[5] Nabbuye-Sekandi J, Makumbi FE, Kasangaki A, Kizza IB, Tugumisirize J, Nshimye E (2011). Patient satisfaction with services in outpatient clinics at Mulago Hospital, Uganda. International Journal for Quality in Healthcare.

[6] Osungbade KO, Shaahu VN, Owoaje EE, Adedokun BO (2013). Patients' Satisfaction with Quality of Anti-Retroviral Services in Central Nigeria: Implications for Strengthening Private Health Services. Journal of Preventive Medicine.

[7] Lochoro P (2004). Measuring patient satisfaction in UCMB health institutions. Health Policy and Development.

[8] Meta and UNHCO (2014). Uganda Client Satisfaction with Services in UGANDA's Public Health Facilities. Medicines Transparency Alliance and Uganda Health Consumers Organization.

[9] Whitworth J, Pickering H, Mulwanyi F, Ruberantwari A, Dolin P, Johnson G Determinants of attendance and patient satisfaction at eye clinics in South-Western Uganda. Health Policy and Planning.

[10] Kwesiga D, Kiwanuka S, Kiwanuka N, Mafigiri D, Kakande N (2013). The Clients' Voice: Satisfaction with HIV/AIDS Care in a Public and Private Health Facility in Kabale District, Uganda. J AIDS Clin Res.

[11] Tetui M, Ekirapa EK, Bua J, Mutebi A, Tweheyo R, Waiswa P (2012). Quality of Antenatal care services in Eastern Uganda: implications for interventions. Pan African Medical Journal.

[12] Ministry of Health (2011). National Health Sector Strategic and Investment Plan, 2011/2014.

[13] Church of Uganda 2015 Diocese.

[14] Olivier J, Tsimpo C, Wodon Q (2012). Satisfaction with faith-inspired health care services in Africa: review and evidence from household surveys.

[15] Ministry of Health (2014). National Health Accounts.

[16] Ministry of Health (2014). Annual Health Sector Performance Report 2013–2014.

[17] Martins JCA (2009). Patients' satisfaction with information on disease and morbidity. Rev Latino-am Enferagen.

[38] Naidu A (2009). “Factors affecting patient satisfaction and healthcare quality”. International Journal of Health Care Quality Assurance.

[19] Shumba CS, Atukunda R, Memiah P (2013). Patient-centred quality care: An assessment of patient involvement. Int J Med Public Health.

[20] Yildirim N, Yildirim H, Mecit SC Exploring the hospital in-patients satisfaction: An analysis on factors, demographics and patient rights.

[21] Ministry of Health (2009). Patients' Charter.

[22] Kiguli J, Ekirapa-kirachoe, Okui O, Mutebi A, macgregor H, Pariyo GW (2009). Increasing access to quality health care for the poor: Community perceptions on quality care in Uganda. Patient Preference and Adherence.

[23] Tumuhamye N, Rutebemberwa E, Kwesiga D, Bagonza J (2013). Client satisfaction with integrated community case management program in Wakiso District, Uganda, October 2012: A cross sectional survey. Health.

